# Elucidation of the Application of Blood Test Biomarkers to Predict Immune-Related Adverse Events in Atezolizumab-Treated NSCLC Patients Using Machine Learning Methods

**DOI:** 10.3389/fimmu.2022.862752

**Published:** 2022-06-30

**Authors:** Jian-Guo Zhou, Ada Hang-Heng Wong, Haitao Wang, Fangya Tan, Xiaofei Chen, Su-Han Jin, Si-Si He, Gang Shen, Yun-Jia Wang, Benjamin Frey, Rainer Fietkau, Markus Hecht, Hu Ma, Udo S. Gaipl

**Affiliations:** ^1^Department of Oncology, The Second Affiliated Hospital of Zunyi Medical University, Zunyi, China; ^2^Translational Radiobiology, Department of Radiation Oncology, Universitätsklinikum Erlangen, Erlangen, Germany; ^3^Department of Radiation Oncology, Universitätsklinikum Erlangen, Erlangen, Germany; ^4^Comprehensive Cancer Center Erlangen-EMN, Erlangen, Germany; ^5^AW Medical Company Limited, Macau, Macau SAR, China; ^6^Thoracic Surgery Branch, National Cancer Institute, National Institutes of Health, Bethesda, MD, United States; ^7^Department of Analytics, Harrisburg University of Science & Technology, Harrisburg, PA, United States; ^8^Department of Biostat & Programming, Sanofi, Bridgewater, NJ, United States; ^9^Department of Orthodontics, School of Stomatology, Zunyi Medical University, Zunyi, China

**Keywords:** blood test, irAE prediction, NSCLC, atezolizumab, machine learning

## Abstract

**Background:**

Development of severe immune-related adverse events (irAEs) is a major predicament to stop treatment with immune checkpoint inhibitors, even though tumor progression is suppressed. However, no effective early phase biomarker has been established to predict irAE until now.

**Method:**

This study retrospectively used the data of four international, multi-center clinical trials to investigate the application of blood test biomarkers to predict irAEs in atezolizumab-treated advanced non-small cell lung cancer (NSCLC) patients. Seven machine learning methods were exploited to dissect the importance score of 21 blood test biomarkers after 1,000 simulations by the training cohort consisting of 80%, 70%, and 60% of the combined cohort with 1,320 eligible patients.

**Results:**

XGBoost and LASSO exhibited the best performance in this study with relatively higher consistency between the training and test cohorts. The best area under the curve (AUC) was obtained by a 10-biomarker panel using the XGBoost method for the 8:2 training:test cohort ratio (training cohort AUC = 0.692, test cohort AUC = 0.681). This panel could be further narrowed down to a three-biomarker panel consisting of C-reactive protein (CRP), platelet-to-lymphocyte ratio (PLR), and thyroid-stimulating hormone (TSH) with a small median AUC difference using the XGBoost method [for the 8:2 training:test cohort ratio, training cohort AUC difference = −0.035 (p < 0.0001), and test cohort AUC difference = 0.001 (p=0.965)].

**Conclusion:**

Blood test biomarkers currently do not have sufficient predictive power to predict irAE development in atezolizumab-treated advanced NSCLC patients. Nevertheless, biomarkers related to adaptive immunity and liver or thyroid dysfunction warrant further investigation.

## Background

Immune checkpoint inhibitor (ICI) therapy has become a widely used first-line therapy for unresectable non-small cell lung cancer (NSCLC) patients. ICIs were developed against programmed cell death ligand 1 (PD-L1) on cancer cells, and the immune suppressive receptors programmed cell death 1 (PD-1) and cytotoxic T-lymphocyte-associated antigen 4 (CTLA-4) on cytotoxic T cells (1). Although ICI therapy can be effective, 5–10% of patients experience immune-related adverse events (irAEs), such as rashes and peripheral neuropathy, as soon as the next day after treatment starts (2–4). Over the course of treatment, some patients may develop more severe symptoms such as pneumonitis, pancreatitis, and vitiligo, which sometimes lead to death. Hence, prediction of severe irAEs before or during early treatment becomes indispensable. In this regard, several studies have been conducted to investigate the correlation between liquid biopsy biomarkers and irAEs (5–7). However, the concurrence of low incidence rate and limited cohort size in many studies restricts the extent of analysis to achieve statistical significance.

The real challenge of irAE prediction is finding the right biomarkers to indicate the immune landscape in a spatial and temporal manner (8). In contrast to therapies with known biological mechanisms or specificity towards certain organs or tissue, targeting of the immune system by ICIs seems to have more unpredictable AEs, particularly irAEs that could potentially affect any part of the body. Nevertheless, research on intrinsic and extrinsic irAE mediators are taking place (8). As such, we reasoned that the prediction of irAEs involves two parallel options: (1) monitoring of immune activity and (2) malfunction detection in vulnerable organs or tissue.

Blood test is regularly conducted before and during treatment and is feasible in almost any hospital. The regular blood test consists of two operations: the blood cell count test (BCT) and the blood biochemistry test (BBT). BCT provides a direct overview of the immune landscape based on the prevalence of immune cell populations. Our previous study established a BCTscore model as a valid predictive and prognostic biomarker for the early prediction of atezolizumab treatment outcomes (9). Because treatment outcome is often correlated to the onset of irAEs (10–12), and pretreatment blood cell count is reported to be associated with pembrolizumab-induced irAEs in patients with advanced NSCLC (13), we hypothesize that BCT biomarkers may also predict irAEs of atezolizumab-treated NSCLC patients. On the other hand, a comprehensive BBT provides a functional overview of various organs. Hence, we investigated the application of blood test parameters to predict irAEs in atezolizumab-treated NSCLC patients using data acquired from the four international, multicenter cohorts of FIR, BIRCH, POPLAR, and OAK.

## Methods

### Study Cohort

Pseudonymized individual participant data from the single-arm phase II studies FIR (NCT01846416) (14) and BIRCH (NCT02031458) (15) and the two-arm randomized controlled trials (RCTs) POPLAR phase II study (NCT01903993) (16) and OAK phase III study (NCT02008227) (17) were provided by Genentech Inc. and accessed through the secure Vivli online platform. Raw data were extracted and compared with the available published data to ensure accuracy. Secondary analysis of the trial data was deemed to be of negligible risk and was approved by the Institutional Review Board of the Second Affiliated Hospital of Zunyi Medical University [No. YXLL (KY-R)-2021-010]. Deidentified data were accessed according to Roche’s policy and process for Vivli. Data analyses were conducted from April 27 to November 30, 2021.

### Definition of irAEs

irAEs are summarized using the National Cancer Institute (NCI) Common Terminology Criteria for Adverse Event (CTCAE) version 4.0 (18) by clinical study. The irAE data were confirmed from the Adverse Events of Special Interest (AESI) dataset according to the Council for International Organisations of Medical Sciences (CIOMS) form. The variable “AEGRP01F = Y” was selected to ensure that the irAE was associated with PD-L1 checkpoint blockade, as already defined by Khan and colleagues (3). Specifically, the CTCAE defines what symptoms constitute AEs and specifically, irAEs. On the other hand, the AESI was compiled by the Vivli platform from which our data were obtained. Incidences of irAE in all four cohorts were obtained under the header “AEGRP01F,” and “Y” stands for “Yes” in the patient records provided by the Vivli platform. We combined the four cohorts into one big cohort comprising 1,320 eligible atezolizumab-treated advanced NSCLC patients with irAE and pretreatment blood test records. After that, we summarized the number of patients with any grade irAE. Because our cohort was assembled from four cohorts, the numbers do not match those which are reported in each individual cohort. This strategy identified a collection of adverse events that had a putative immune-related etiology.

### Machine Learning Methods

Because of the complexity of different parameters obtained from BCT and BBT, machine learning was used instead of conventional Cox regression model. Here, we applied seven machine learning methods (**Supplementary Figure S1**). The methods used in this study include the following: (1, 2) the Lasso (LASSO) or Elastic-Net Regularized Generalized Linear Model (GLM) (R package glmnet v.4.1.3) (19), (3) the Support Vector Machines model (SVM; R package e1071 v.1.7.9) (20), (4) the Recursive Partitioning and Regression Trees model, also known as Decision Tree model (DT; R package rpart v. 4.1.15) (21), (5) the Random Forest model (RF; R package randomForest v. 4.6.14) (22), (6) the eXtreme Gradient Boosting model (XGB; R package xgboost v. 0.4.2) (23), and (7) the Generalized Boosted Regression Models (GBMs; R package gbm v.2.1.8) (24). The function *createDataPartition* of *caret* (Classification and Regression Training) package v.6.0.89 (25) was used to create balanced splits of the data as training and test cohorts.

### Analytic Procedures

**Figure 1:**
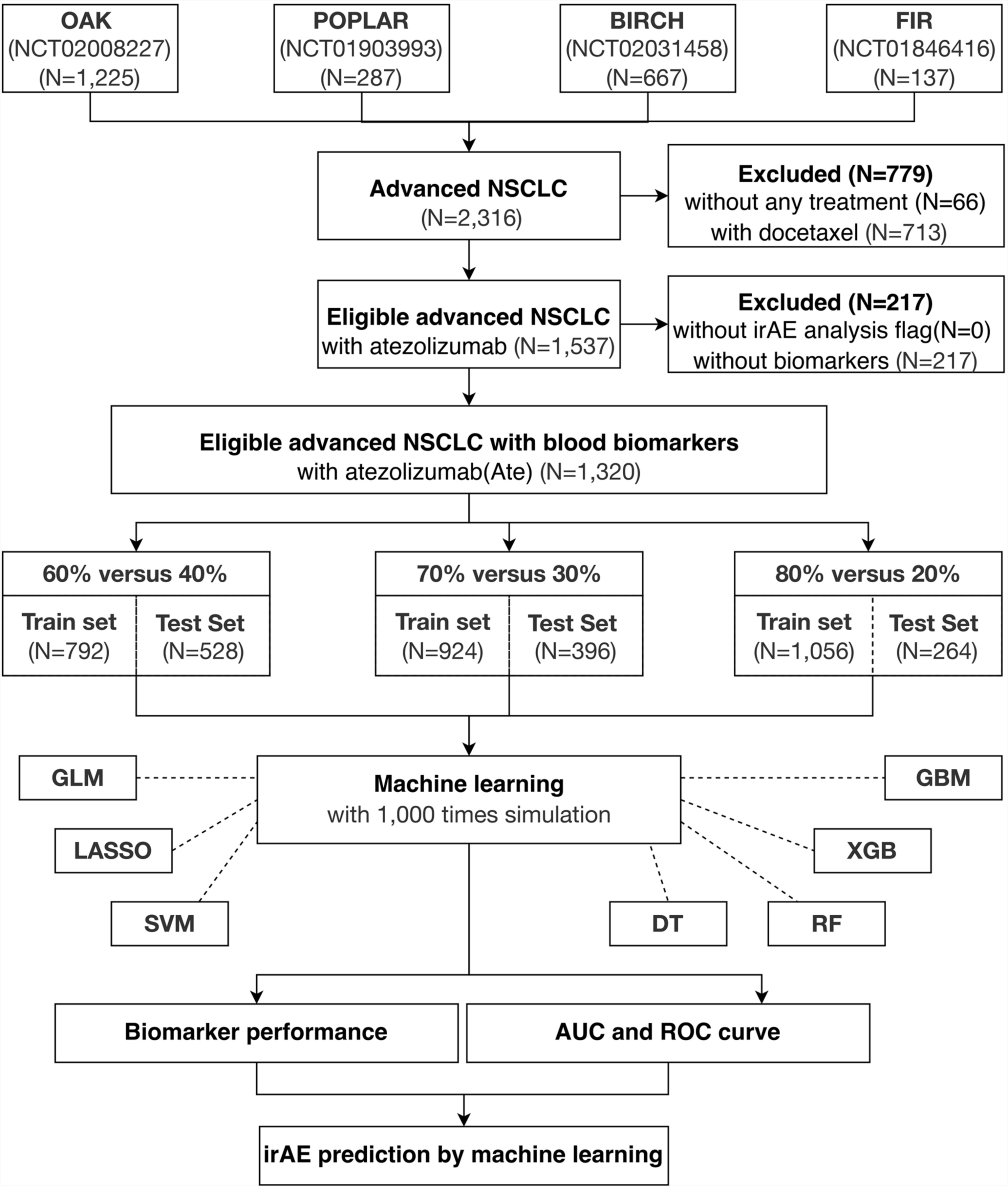
Study overview. A total of 1,320 eligible NSCLC patients undergoing atezolizumab single-agent treatment is obtained from four international, multicenter clinical trials for this study.

The paradigm of this study is illustrated in **Figure 1**. First, all four cohorts containing 2,316 advanced NSCLC patients were combined, of which 1,537 were eligible atezolizumab-treated advanced NSCLC patients with irAE. A total of 1,320 eligible atezolizumab-treated advanced NSCLC patients with irAE and their pretreatment blood test records were randomly separated into training and test cohorts under the criterion that each sample population contained 5% patients displaying any form of irAE. Next, blood test parameters with >10% missing values in the sample population were removed from analysis. Consequently, a total of 21 blood test parameters were fed into the machine learning models as primary classifiers. Binary outcomes of any irAE was applied to the prediction models. The sample populations for the training and test cohorts were randomly selected at the ratios of 6:4, 7:3, and 8:2 from the combined population of the four clinical trials for 1,000 times simulation. The blood test parameters were evaluated by the importance score generated by each method after each simulation for their performance. Model performance was evaluated by the area under curve (AUC) and corresponding 95% confidence interval (CI) of the receiver operating characteristic (ROC) curve. Analysis of variance (ANOVA) and Tukey honestly significant difference (Tukey HSD) tests were performed by R base package. Sensitivity, specificity, accuracy, and the Kappa statistic were calculated by ROCR package (v.1.0-11) (26) and interpreted as previously described (27).

## Results

### Blood Test Biomarkers That Indicate Adaptive Immunity and Liver Function Are Useful for irAE Prediction in Atezolizumab-Treated Advanced NSCLC Patients

**Figure 2:**
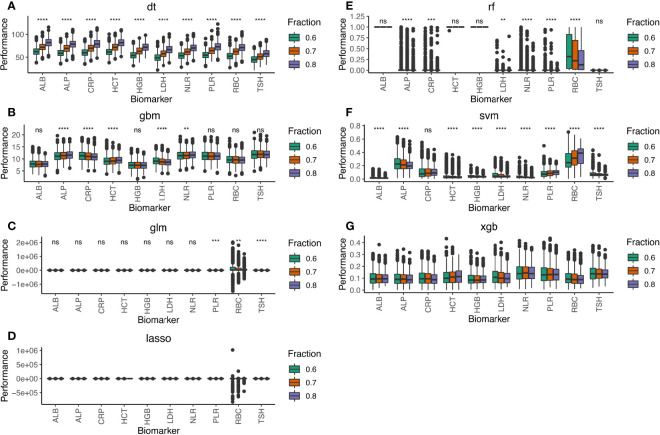
Performance of the 10-biomarker panel evaluated by the seven machine learning methods of **(A)** DT, **(B)** GBM, **(C)** GLM, **(D)** LASSO, **(E)** RF, **(F)** SVM, and **(G)** XGB. Performance scores were computed by each machine learning method for 1,000 simulations of the training and test datasets at 8:2, 7:3, and 6:4 cohort ratios randomly selected from the combined cohort comprising 1,320 atezolizumab-treated and whisker plot shows the median (thick black line in the middle of the box), the interquartile range between 75% and 25% (upper and lower end of the box), and 1.5 multiplied by upper or lower interquartile range (whiskers), respectively. ns is P ≥ 0.05, *P < 0.05, **P < 0.01, *** P < 0.001, ****P < 0.0001.

Initially, we compared the median performance of all 21 blood test biomarkers after 1,000 simulations on the training cohorts by the seven machine learning methods (**Supplementary Figure S2**). We selected the top 10 biomarkers that stably displayed above median performance in all simulations (**Figure 2**). Among these biomarkers were the neutrophil-to-lymphocyte ratio (NLR) and platelet-to-lymphocyte ratio (PLR). NLR and PLR indicate the host’s immune landscape and had been selected for our previous BCTscore model to predict survival benefit (9). Alternatively, red blood cell count (RBC), hematocrit (HCT), hemoglobin (HGB), albumin (ALB), and alkaline phosphatase (ALP) that indicate liver function also demonstrated good performance in irAE prediction. On the other hand, the BBT biomarkers of lactate dehydrogenase (LDH) and C-reactive protein (CRP) that indicate tissue damage and infection performed well in our first biomarker screening. Additionally, the BBT biomarker of thyroid-stimulating hormone (TSH) that indirectly suggest infection and cancer also demonstrated good performance in irAE prediction. Among the 10 biomarkers, the top 3 blood test biomarkers were PLR, CRP, and TSH (**Supplementary Figure S3**). Results showed that the 10-biomarker panel is most optimal for irAE prediction, where the AUC of the test cohort differed insignificantly from the training cohort at all three cohort ratios. However, the three-biomarker panel consisting of PLR, CRP, and TSH is sufficient, with small AUC difference from the 10-biomarker panel [for 8:2 training:test cohort ratio, the mean difference in AUC of the 10-biomarker panel vs. 3-biomarker panel of LASSO: training = −0.044 (p<0.0001), test = −0.026 (p<0.0001); and XGB: training = −0.035 (p<0.0001), test = 0.001 (p=0.965)] (**Supplementary Table S2**).

Taken together, we conclude that blood test biomarkers indicating adaptive immunity and liver or thyroid dysfunction are useful for irAE prediction in atezolizumab-treated advanced NSCLC patients.

### LASSO and XGB Exhibited the Best Performance in irAE Prediction in This Study

Because the seven machine learning methods are vastly different in terms of mathematical modeling and application, we tested which method is optimal for irAE prediction in atezolizumab-treated advanced NSCLC patients. Therefore, we compared the AUC distribution of our retrospective cohorts using the 21-, 10-, and 3-biomarker panels, respectively (**Supplementary Figure S4**). Furthermore, the combined cohort was randomly separated into training and test cohorts at different ratios to evaluate the consistency of irAE prediction in a retrospective manner. Results showed that increasing the training:test cohort ratio improves the predictive power of all seven machine learning methods. Furthermore, the 21-biomarker panel generally yields higher median AUC as compared to the 10- and 3-biomarker panels. Nevertheless, the seven machine learning methods displayed fundamental differences in irAE prediction performance. For instance, although RF consistently demonstrated median AUC >0.8 in the 1,000 simulations of the training cohorts comprising of 80%, 70%, and 60% of the combined cohort, its performance on the test cohort did not significantly differ from the other machine learning methods; the median AUC of all seven machine learning methods lay in the range of 0.517–0.581 throughout the 1,000 simulations of the test cohorts. Similarly, the DT and SVM models suffered from discrepancy between the training and test cohort prediction like the RF method. In contrast, the unsupervised, linear GLM and GBM models displayed consistent predictive power between training and test cohorts and yielded comparative median AUC to LASSO. However, the bigger variance in AUC distribution is less favorable for their application in real-life, prospective predictions.

**Figure 3:**
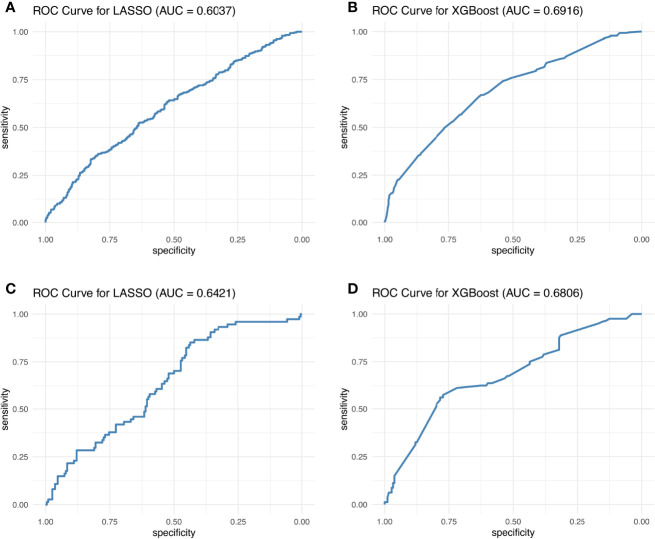
Best ROC curves of the 10-biomarker panel evaluated by the LASSO and XGB methods. The best ROC curves were obtained at 8:2 cohort ratio of the training and test datasets by the LASSO and XGB methods.

Hence, LASSO and XGB showed the highest potential as optimal irAE prediction methods. These two methods gave the best AUC for the 10-biomarker panel (for 8:2 training:test cohort ratio, the mean AUC of the 10-biomarkers panel calculated by LASSO: training = 0.604, test = 0.642; and XGB: training = 0.692, test = 0.681) (**Figure 3**). Other reasons for choosing these two methods include the following: (1) LASSO and XGB depicted good predictive power in both training and test cohorts at all three cohort ratios (**Supplementary Figure S4**); (2) they were minimally affected by reducing the number of biomarkers (**Table 1**) as compared to the other methods (**Supplementary Table S1**); and (3) these two methods are fundamentally different—LASSO uses a supervised, linear algorithm, whereas XGB uses a supervised, non-linear algorithm.

**Table 1:** Median AUC distribution of the three blood test biomarker panels.

	Cohort ratio	21-Biomarker		10-Biomarker		3-Biomarker	
Method	(training:test)	Training	Test	Training	Test	Training	Test
**LASSO**	6:4	0.591 ± 0.027	0.574 ± 0.023	0.591 ± 0.024	0.578 ± 0.022	0.556 ± 0.021	0.553 ± 0.024
	7:3	0.594 ± 0.027	0.575 ± 0.027	0.594 ± 0.025	0.579 ± 0.027	0.557 ± 0.022	0.554 ± 0.028
	8:2	0.596 ± 0.030	0.578 ± 0.036	0.597 ± 0.029	0.582 ± 0.037	0.557 ± 0.026	0.556 ± 0.035
**XGB**	6:4	0.662 ± 0.089	0.563 ± 0.023	0.662 ± 0.080	0.564 ± 0.023	0.633 ± 0.062	0.561 ± 0.023
	7:3	0.670 ± 0.083	0.564 ± 0.028	0.664 ± 0.075	0.565 ± 0.028	0.639 ± 0.057	0.565 ± 0.027
	8:2	0.684 ± 0.079	0.566 ± 0.035	0.670 ± 0.070	0.567 ± 0.034	0.642 ± 0.054	0.571 ± 0.035

**Figure 3** displays the best AUC obtained from LASSO and XGB among 1,000 simulations for the training and test cohorts, whereas in **Table 1** and **Supplementary Figure S4**, the AUC distribution of the 1,000 simulations are summarized. One can therefore conclude that the AUC is still unsatisfactory for real-life application. This conclusion is reinforced by the observed Kappa statistic (**Supplementary Table S3**). The low Kappa statistic suggested that the accuracy of prediction is unacceptable for all three blood test biomarker panels. Hence, we concluded that blood test biomarkers do not have sufficient predictive power to predict irAE development in atezolizumab-treated advanced NSCLC patients.

## Discussion

In this study, we analyzed 21 blood test biomarkers with comparison of the seven machine learning methods to identify the optimal biomarker panel and machine learning methods for irAE prediction in a combined cohort from four retrospective, multi-center clinical trials, involving advanced NSCLC patients treated with the anti-PD-L1 atezolizumab. Results showed that blood test biomarkers do not have sufficient predictive power to predict irAE development in atezolizumab-treated advanced NSCLC patients.

Better biomarkers are urgently needed. A literature review of the biological mechanisms of the best-performing biomarkers showed that the liver is critical for adaptive immunity (28–30) and is the primary synthetic site for CRP (31). Hence, although none of the biomarkers related to liver function made to the top three-biomarker panel, the inclusion of these biomarkers in the 10-biomarker panel increased median AUC of irAE prediction from that of the three-biomarker panel, with small but significant difference in the training cohort.

In the three-biomarker panel, PLR indisputably correlates with survival outcomes during ICI therapy (32). However, CRP and TSH are less studied. CRP has long been used as a universal biomarker for infection-induced inflammation (33). However, it has only been recently reported that different isoforms of CRP harness different biological pathways to trigger inflammation (31), while our clinical blood tests merely detect naive CRP. Nonetheless, the CRP/albumin ratio has been reported to be positively correlated with PLR and could serve as an independent risk factor for overall survival (OS) in advanced NSCLC patients (34, 35). Hence, although the biological mechanism remains elusive, CRP depicted good performance for irAE prediction in NSCLC, in consistence to previous findings in melanoma patients (36). On the other hand, even though the mechanism of TSH is also unclear, thyroid dysfunction is a prevalent irAE in NSCLC patients treated with the anti-PD1 antibody nivolumab (37) and thus is not surprising to perform well in this study.

Khan and colleagues found that genetic variation that is associated with thyroid autoimmunity interacts with biological pathways driving the systemic immune response to ICI (2). Another study demonstrated that activated CD4 memory T-cell abundance and TCR diversity are associated with severe irAE development regardless of the organ system involvement (38). Collectively, we deduce that biomarkers related to adaptive immunity and liver or thyroid dysfunction warrants further investigation.

Notably, there was insignificant difference in irAE prediction in the test cohorts for all seven machine learning methods, no matter how well these methods performed in the training cohorts. This observation suggested that shuffling patients or adjusting the training:test cohort ratios did not improve the irAE prediction model’s performance. Hence, the machine learning methods of RF, DT, and SVM were likely overfittings during training, thus performing poorly in the test cohorts because all patients differed between the training and test cohorts, except the fact that both cohorts contained 5% patients exhibiting any form of irAE. Additionally, 1,000 simulations of random selection of 1,320 patients into the training and test cohorts at different ratios ruled out the possibility of sample bias. Avoiding sample bias is particularly important because of the different patient eligibility criteria of the four clinical trials: specifically, except that the BIRCH and FIR trials contained a fraction of PD-L1-positive (PD-L1 ≥ 5%) advanced NSCLC patients with no prior chemotherapy, all trials recruited advanced NSCLC patients who underwent platinum-based therapy. Therefore, the failure of certain methods to predict irAE could not stem from accidental sample bias during simulation. Furthermore, it is noteworthy that the difference in median AUC predicted by the seven machine learning methods using the top 3-biomarker panel (**Supplementary Figure S3**) narrowed as compared to the 10- and 21-biomarker panels (**Supplementary Figure S2**). Considering that these methods exploit disparate mathematical algorithms, it became explicit that the nature of algorithm did not affect the conclusion. Nevertheless, as pointed out earlier, overfitting seemed to be more prevalent in some algorithms than others. Moreover, some biomarkers with widely variable absolute detected range among individual patients may show inconsistent performance in some methods. For example, RBC showed a wide range of performance in the linear models of LASSO and GLM (**Supplementary Figure S2**). However, the fact that RBC performance was more consistent in the other linear models of SVM, DT, and GBM suggested that the divergence was method specific. In contrast, PLR is also inherently divergent like RBC, but its performance was much more consistent in all methods. Hence, even though blood test biomarkers may exhibit variable detected ranges, their irAE prediction performance is relatively stable. Therefore, caution should be taken when assessing the performance variance of certain biomarkers as a criterion for biomarker selection. In-depth analysis of the common detectable range and biological mechanism of each biomarker is highly recommended during panel construction.

## Conclusion

Blood test biomarkers do not have sufficient predictive power to predict irAE development in atezolizumab-treated advanced NSCLC patients. Biomarkers related to adaptive immunity and liver or thyroid dysfunction warrant further investigation.

## Data Availability Statement

The datasets presented in this study can be found in online repositories. The names of the repository/repositories and accession number(s) can be found below: this publication is based on research using data from data contributors, Roche, that have been made available through Vivli, Inc. (Data Request ID: 5935; Lead Investigator: J-GZ). Vivli has not contributed to or approved, and is not in any way responsible for, the contents of this publication.

## Ethics Statement

The studies involving human participants were reviewed and approved by the Institutional Review Board of the Second Affiliated Hospital of Zunyi Medical University [No. YXLL(KY-R)-2021-010]. Written informed consent for participation was not required for this study in accordance with the national legislation and the institutional requirements.

## Author Contributions

Conceptualization: JGZ, AHW, HM, UG, and SHJ. Methodology: JGZ, AHW, and FT. Validation: MH, HM, UG, and JGZ. Data curation: JGZ, AHW, GS, SSH, HM, SRC, and UG. Writing—original draft preparation: AHW, HW, and JGZ. Writing—review and editing: UG, HM, BF and MH. Visualization: JGZ and HW. Supervision: HM, UG, and JGZ. Project administration: JGZ, HM, and UG. Funding acquisition: JGZ, SHJ, and AHW. All authors contributed to thearticle and approved the submitted version.

## Funding

This research was funded by the National Natural Science Foundation of China (Grant No. 82060475), the National Natural Science Foundation of Guizhou Province (Grant Nos. [2020]1Z062, ZK2021-YB435, and ZK2022-YB632), and Lian Yun Gang Shi Hui Lan Public Foundation (Grant No. HL-HS2020-92). AW Medical Company Limited received startup funding from the University Development Fund of University of Macau.

## Conflict of Interest

AHW is a founder and shareholder of AW Medical Company Limited. MH reports collaborations with Merck Serono (advisory role, speakers’ bureau, honoraria, travel expenses, and research funding), MSD (advisory role, speakers’ bureau, honoraria, travel expenses, and research funding), AstraZeneca (research funding), Novartis (research funding), BMS (advisory role, honoraria, and speakers’ bureau), and Teva (travel expenses). UG and RF received support for presentation activities for Dr Sennewald Medizintechnik GmbH, have received support for investigator initiated clinical studies (IITs) from MSD and AstraZeneca, and contributed at Advisory Boards Meetings of AstraZeneca and Bristol-Myers Squibb.

The remaining authors declare that the research was conducted in the absence of any commercial or financial relationships that could be construed as a potential conflict of interest.

## Publisher’s Note

All claims expressed in this article are solely those of the authors and do not necessarily represent those of their affiliated organizations, or those of the publisher, the editors and the reviewers. Any product that may be evaluated in this article, or claim that may be made by its manufacturer, is not guaranteed or endorsed by the publisher.

## Supplementary Material

Supplementary Figure 1 Graphical comparison of the 7 machine learning methods used in this study.

Supplementary Figure 2 Performance of the 21-biomarkers panel evaluated by the 7 ML methods. Performance scores were computed by each machine learning method for 1,000 simulations of the training and test datasets at 8:2, 7:3 and 6:4 cohort ratios randomly selected from the combined cohort comprising 1,320 atezolizumab-treated NSCLC patients. The box and whisker plot shows the median (thick black line in the middle of the box), the interquantile range between 75% and 25% (upper and lower end of the box), and 1.5 * upper or lower interquantile range (whiskers), respectively.

Supplementary Figure 3 Performance of the 3-biomarkers panel evaluated by 7 ML methods. Performance scores were computed by each machine learning method for 1,000 simulations of the training and test datasets at 8:2, 7:3 and 6:4 cohort ratios randomly selected from the combined cohort comprising 1,320 atezolizumab-treated NSCLC patients. The box and whisker plot shows the median (thick black line in the middle of the box), the interquantile range between 75% and 25% (upper and lower end of the box), and 1.5 * upper or lower interquantile range (whiskers), respectively.

Supplementary Figure 4 AUC distribution of the 3 biomarker panels of this study. AUC was calculated by 7 machine learning methods from 1,000 simulations of the training and test datasets at 8:2, 7:3 and 6:4 cohort ratios randomly selected from the combined cohort comprising 1,320 atezolizumab-treated NSCLC patients. The box and whisker plot shows the median (thick black line in the middle of the box), the interquantile range between 75% and 25% (upper and lower end of the box), and 1.5 * upper or lower interquantile range (whiskers), respectively.

Supplementary Table 1 Summary of AUC distribution. AUC was calculated by 7 machine learning methods from 1,000 simulations of the training and test datasets at 8:2, 7:3 and 6:4 cohort ratios randomly selected from the combined cohort comprising 1,320 atezolizumab-treated NSCLC patients.

Supplementary Table 2 Summary of ANOVA and Tukey HSD tests of the 3 biomarker panels of this study. ANOVA and Tukey HSD tests were performed on the AUC calculated by 7 machine learning methods from 1,000 simulations of the training and test datasets at 8:2, 7:3 and 6:4 cohort ratios randomly selected from the combined cohort comprising 1,320 atezolizumab-treated NSCLC patients.

Supplementary Table 3 Summary of assay performance. Sensitivity, specificity, accuracy, and the kappa statistic were computed for the 7 machine learning methods from 1,000 simulations of the training and test datasets at 8:2, 7:3 and 6:4 cohort ratios randomly selected from the combined cohort comprising 1,320 atezolizumab-treated NSCLC patients.

## References

[1] Duma N Santana-Davila R Molina JR. Non–Small Cell Lung Cancer: Epidemiology, Screening, Diagnosis, and Treatment. Mayo Clinic Proc (2019) 94(8):1623–40. doi: 10.1016/j.mayocp.2019.01.013 31378236

[2] Khan Z Hammer C Carroll J Di Nucci F Acosta SL Maiya V. Genetic Variation Associated With Thyroid Autoimmunity Shapes the Systemic Immune Response to PD-1 Checkpoint Blockade. Nat Commun (2021) 12(1):3355. doi: 10.1038/s41467-021-23661-4 34099659 PMC8184890

[3] Khan Z Di Nucci F Kwan A Hammer C Mariathasan S Rouilly V. Polygenic Risk for Skin Autoimmunity Impacts Immune Checkpoint Blockade in Bladder Cancer. Proc Natl Acad Sci (2020) 117(22):12288. doi: 10.1073/pnas.1922867117 32430334 PMC7275757

[4] Martins F Sofiya L Sykiotis GP Lamine F Maillard M Fraga M. Adverse Effects of Immune-Checkpoint Inhibitors: Epidemiology, Management and Surveillance. Nat Rev Clin Oncol (2019) 16(9):563–80. doi: 10.1038/s41571-019-0218-0 31092901

[5] Honrubia-Peris B Garde-Noguera J García-Sánchez J Piera-Molons N Llombart-Cussac A Fernández-Murga ML. Soluble Biomarkers With Prognostic and Predictive Value in Advanced Non-Small Cell Lung Cancer Treated With Immunotherapy. Cancers (Basel) (2021) 13(17):4280. doi: 10.3390/cancers13174280 34503087 PMC8428366

[6] Indini A Rijavec E Grossi F. Circulating Biomarkers of Response and Toxicity of Immunotherapy in Advanced Non-Small Cell Lung Cancer (NSCLC): A Comprehensive Review. Cancers (Basel) (2021) 13(8):1794. doi: 10.3390/cancers13081794 33918661 PMC8070633

[7] Pavan A Calvetti L Dal Maso A Attili I Del Bianco P Pasello G. Peripheral Blood Markers Identify Risk of Immune-Related Toxicity in Advanced Non-Small Cell Lung Cancer Treated With Immune-Checkpoint Inhibitors. Oncologist (2019) 24(8):1128–36. doi: 10.1634/theoncologist.2018-0563 PMC6693718 31015312

[8] Bayless NL Bluestone JA Bucktrout S Butterfield LH Jaffee EM Koch CA. Development of Preclinical and Clinical Models for Immune-Related Adverse Events Following Checkpoint Immunotherapy: A Perspective From SITC and AACR. J ImmunoTher Cancer (2021) 9(9):e002627. doi: 10.1136/jitc-2021-002627 34479924 PMC8420733

[9] Zhou J-G Wong AH-H Wang H Jin S-H Tan F Chen Y-Z. 329 Early Blood Cell Count Test (BCT) for Survival Prediction for non-Small Cell Lung Cancer Patients Treated With Atezolizumab: Integrated Analysis of 4 Multicenter Clinical Trials. J ImmunoTher Cancer (2021) 9(2):A355–5. doi: 10.1136/jitc-2021-SITC2021.329

[10] Schweizer C Schubert P Rutzner S Eckstein M Haderlein M Lettmaier S. Prospective Evaluation of the Prognostic Value of Immune-Related Adverse Events in Patients With non-Melanoma Solid Tumour Treated With PD-1/PD-L1 Inhibitors Alone and in Combination With Radiotherapy. Eur J Cancer (2020) 140:55–62. doi: 10.1016/j.ejca.2020.09.001 33045663

[11] Eggermont AMM Kicinski M Blank CU Mandala M Long GV Atkinson V. Association Between Immune-Related Adverse Events and Recurrence-Free Survival Among Patients With Stage III Melanoma Randomized to Receive Pembrolizumab or Placebo: A Secondary Analysis of a Randomized Clinical Trial. JAMA Oncol (2020) 6(4):519–27. doi: 10.1001/jamaoncol.2019.5570 PMC6990933 31895407

[12] Teraoka S Fujimoto D Morimoto T Kawachi H Ito M Sato Y. Early Immune-Related Adverse Events and Association With Outcome in Advanced Non-Small Cell Lung Cancer Patients Treated With Nivolumab: A Prospective Cohort Study. J Thorac Oncol (2017) 12(12):1798–805. doi: 10.1016/j.jtho.2017.08.022 28939128

[13] Egami S Kawazoe H Hashimoto H Uozumi R Arami T Sakiyama N. Peripheral Blood Biomarkers Predict Immune-Related Adverse Events in Non-Small Cell Lung Cancer Patients Treated With Pembrolizumab: A Multicenter Retrospective Study. J Cancer (2021) 12(7):2105–12. doi: 10.7150/jca.53242 PMC7974524 33754009

[14] Spigel DR Chaft JE Gettinger S Chao BH Dirix L Schmid P. FIR: Efficacy, Safety, and Biomarker Analysis of a Phase II Open-Label Study of Atezolizumab in PD-L1–Selected Patients With NSCLC. J Thorac Oncol (2018) 13(11):1733–42. doi: 10.1016/j.jtho.2018.05.004 PMC7455890 29775807

[15] Peters S Gettinger S Johnson ML Jänne PA Garassino MC Christoph D. Phase II Trial of Atezolizumab As First-Line or Subsequent Therapy for Patients With Programmed Death-Ligand 1–Selected Advanced Non–Small-Cell Lung Cancer (BIRCH). J Clin Oncol (2017) 35(24):2781–9. doi: 10.1200/JCO.2016.71.9476 PMC5562171 28609226

[16] Fehrenbacher L Spira A Ballinger M Kowanetz M Vansteenkiste J Mazieres J. Atezolizumab Versus Docetaxel for Patients With Previously Treated non-Small-Cell Lung Cancer (POPLAR): A Multicentre, Open-Label, Phase 2 Randomised Controlled Trial. Lancet (2016) 387(10030):1837–46. doi: 10.1016/S0140-6736(16)00587-0 26970723

[17] Rittmeyer A Barlesi F Waterkamp D Park K Ciardiello F von Pawel J. Atezolizumab Versus Docetaxel in Patients With Previously Treated Non-Small-Cell Lung Cancer (OAK): A Phase 3, Open-Label, Multicentre Randomised Controlled Trial. Lancet (2017) 389(10066):255–65. doi: 10.1016/S0140-6736(16)32517-X PMC6886121 27979383

[18] SERVICES USDOHAH. Common Terminology Criteria for Adverse Events (CTCAE) Version 4.0. 2009 03.30. Available at: https://evs.nci.nih.gov/ftp1/CTCAE/CTCAE_4.03/Archive/CTCAE_4.0_2009-05-29_QuickReference_8.5x11.pdf.

[19] Friedman JH Hastie T Tibshirani R. Regularization Paths for Generalized Linear Models *via* Coordinate Descent. J Stat Software (2010) 33(1):1–22. doi: 10.18637/jss.v033.i01 PMC2929880 20808728

[20] David M Evgenia D Kurt H Andreas W Friedrich L. Package ‘E1071’. (2022).

[21] Terry T Beth A Brian R. Package ‘Rpart’. (2022).

[22] Breiman L Cutler A Liaw A Wiener M. Package ‘Randomforest’. (2022).

[23] Chen T He T Benesty M Khotilovich V Tang Y Cho H. Xgboost: Extreme Gradient Boosting, Vol. 1. (2015). pp. 1–4, R package version 04-2.

[24] Greenwell B Boehmke B Cunningham J Developers G. Package ‘Gbm’. (2022).

[25] Kuhn M Wing J Weston S Williams A Keefer C Engelhardt A. Package ‘Caret’. (2022).

[26] Sing T Sander O Beerenwinkel N Lengauer T. ROCR: Visualizing Classifier Performance in R. Bioinformatics (2005) 21(20):3940–1. doi: 10.1093/bioinformatics/bti623 16096348

[27] Feuerman M Miller AR. The Kappa Statistic as a Function of Sensitivity and Specificity. Int J Math Educ Sci Technol (2005) 36(5):517–27. doi: 10.1080/00207390500063967

[28] Crispe IN. The Liver as a Lymphoid Organ. Annu Rev Immunol (2009) 27:147–63. doi: 10.1146/annurev.immunol.021908.132629 19302037

[29] Zheng M Tian Z. Liver-Mediated Adaptive Immune Tolerance. Front Immunol (2019) 10:2525. doi: 10.3389/fimmu.2019.02525 31787967 PMC6856635

[30] Jenne CN Kubes P. Immune Surveillance by the Liver. Nat Immunol (2013) 14(10):996–1006. doi: 10.1038/ni.2691 24048121

[31] Sproston NR Ashworth JJ. Role of C-Reactive Protein at Sites of Inflammation and Infection. Front Immunol (2018) 9:754. doi: 10.3389/fimmu.2018.00754 29706967 PMC5908901

[32] Qi W-X Xiang Y Zhao S Chen J. Assessment of Systematic Inflammatory and Nutritional Indexes in Extensive-Stage Small-Cell Lung Cancer Treated With First-Line Chemotherapy and Atezolizumab. Cancer Immunol Immunother (2021) 70(11):3199–206. doi: 10.1007/s00262-021-02926-3 PMC10991671 33796915

[33] Black S Kushner I Samols D. C-Reactive Protein. J Biol Chem (2004) 279(47):48487–90. doi: 10.1074/jbc.R400025200 15337754

[34] Ni XF Wu J Ji M Shao YJ Xu B Jiang JT. Effect of C-Reactive Protein/Albumin Ratio on Prognosis in Advanced Non-Small-Cell Lung Cancer. Asia Pac J Clin Oncol (2018) 14(6):402–9. doi: 10.1111/ajco.13055 30178541

[35] Zhou J-G Ma H Gaipl U Frey B Hecht M Fietkau R. Abstract 382: Longitudinal C-Reactive Protein (CRP) as an Individualized Dynamic Predictor for Metastatic Cancer Patients Treated With Immune Checkpoint Inhibitors: Findings From the Prospective ST-ICI Cohort. Cancer Res (2021) 81(13):382. doi: 10.1158/1538-7445.AM2021-382

[36] Lauwyck J Beckwée A Santens A Schwarze JK Awada G Vandersleyen V. C-Reactive Protein as a Biomarker for Immune-Related Adverse Events in Melanoma Patients Treated With Immune Checkpoint Inhibitors in the Adjuvant Setting. Melanoma Res (2021) 31:371–7. doi: 10.1097/cmr.0000000000000748 34054056

[37] Sato K Akamatsu H Murakami E Sasaki S Kanai K Hayata A. Correlation Between Immune-Related Adverse Events and Efficacy in non-Small Cell Lung Cancer Treated With Nivolumab. Lung Cancer (2018) 115:71–4. doi: 10.1016/j.lungcan.2017.11.019 29290265

[38] Lozano AX Chaudhuri AA Nene A Bacchiocchi A Earland N Vesely MD. T Cell Characteristics Associated With Toxicity to Immune Checkpoint Blockade in Patients With Melanoma. Nat Med (2022) 28(2):353–62. doi: 10.1038/s41591-021-01623-z PMC8866214 35027754

