# Supplementary figures and images for: Elucidation of the Application of Blood Test Biomarkers to Predict Immune-Related Adverse Events in Atezolizumab-Treated NSCLC Patients Using Machine Learning Methods
Source: Front Immunol. 2022 Jun 30;13:862752. doi: 10.3389/fimmu.2022.862752 (PMC9284319; doi:10.3389/fimmu.2022.862752)

- Comparison of linear and non-linear prediction models

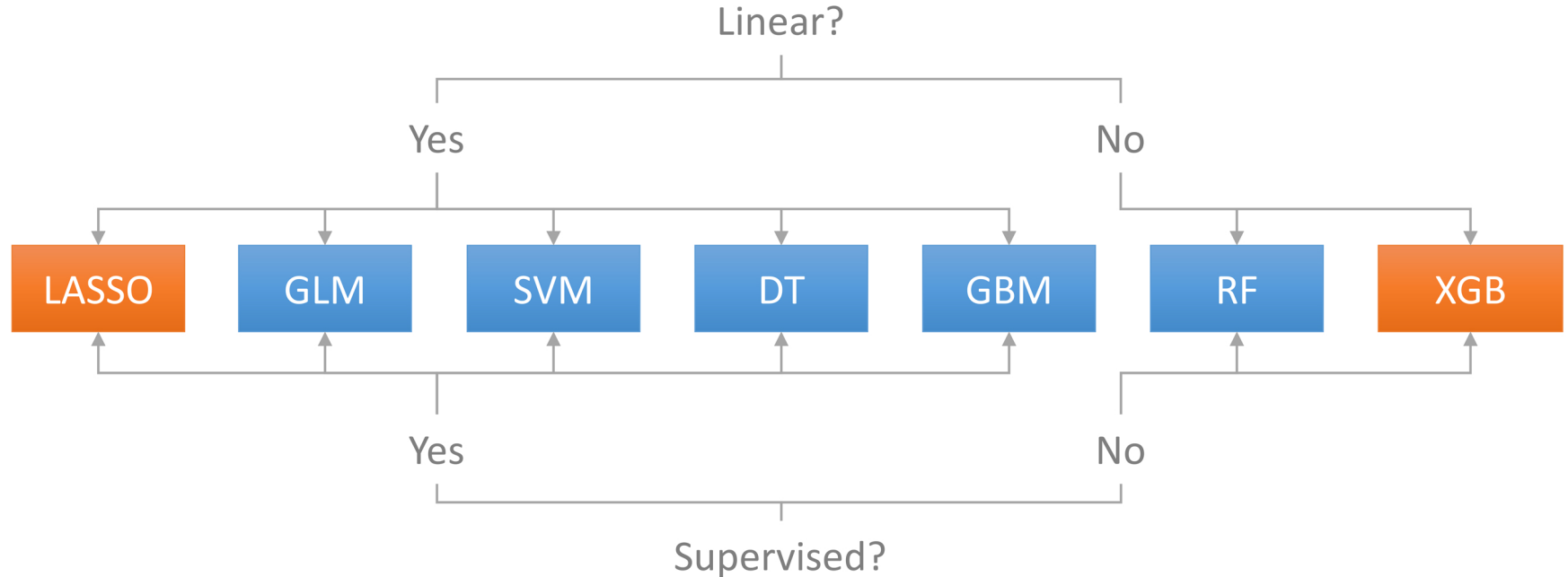

Supplement: Supplementary Figure 1 — Graphical comparison of the 7 machine learning methods used in this study. [file DataSheet_1.pdf]

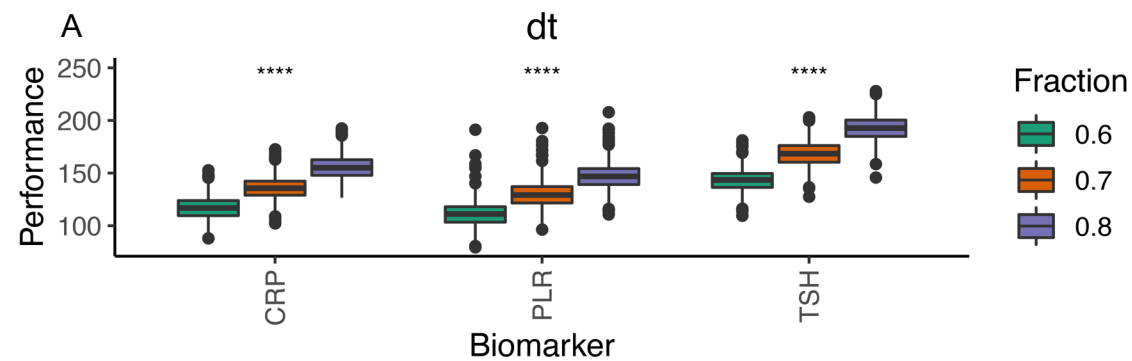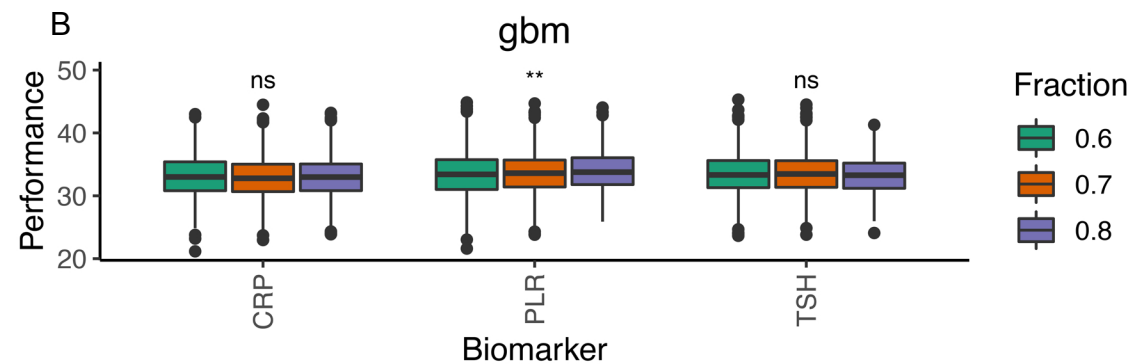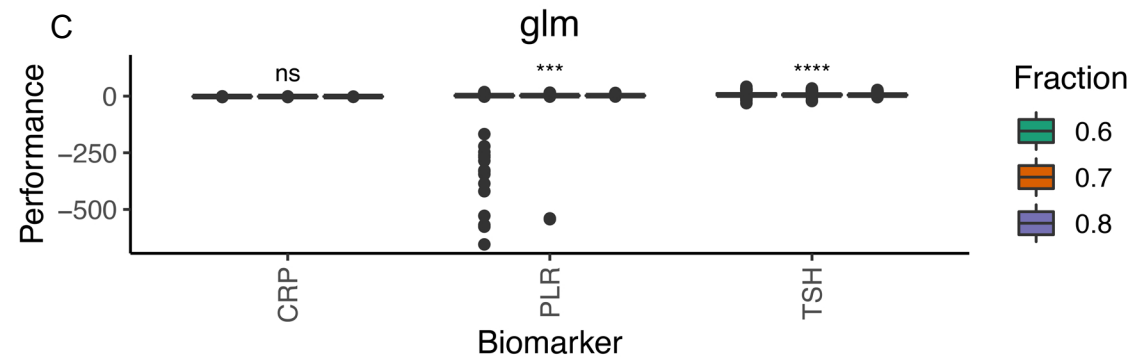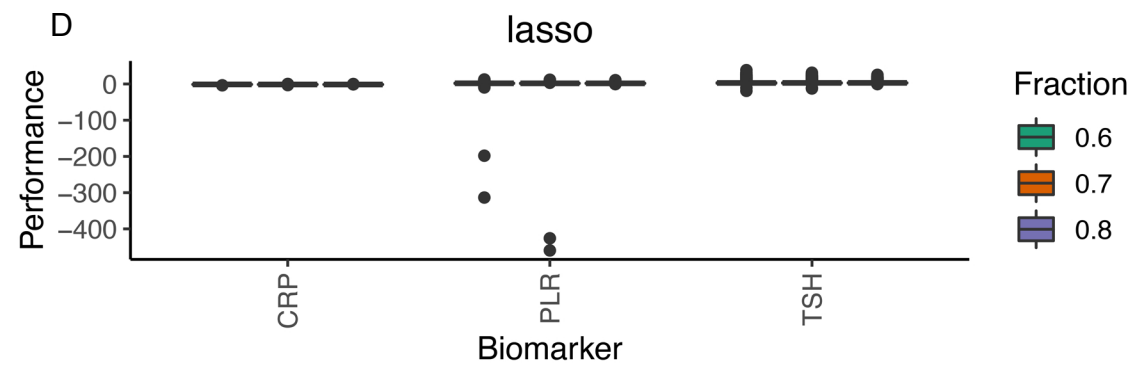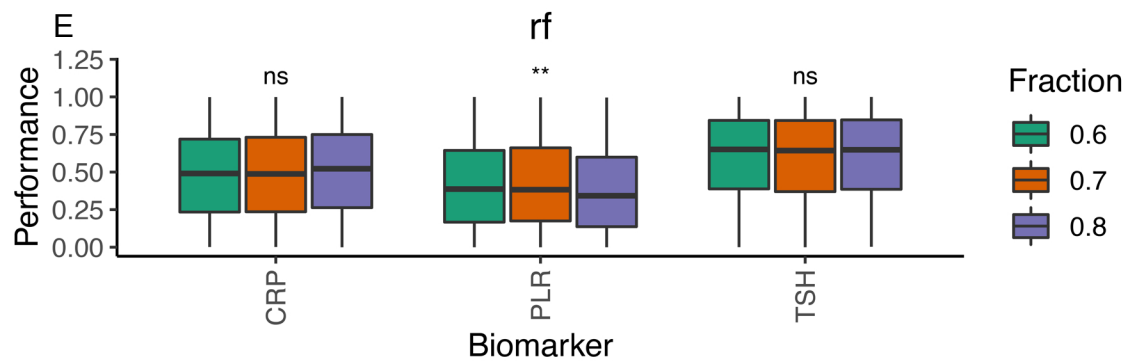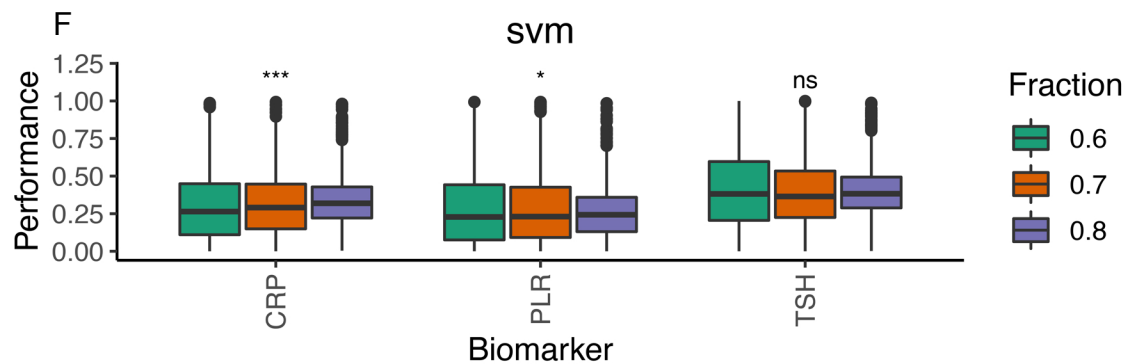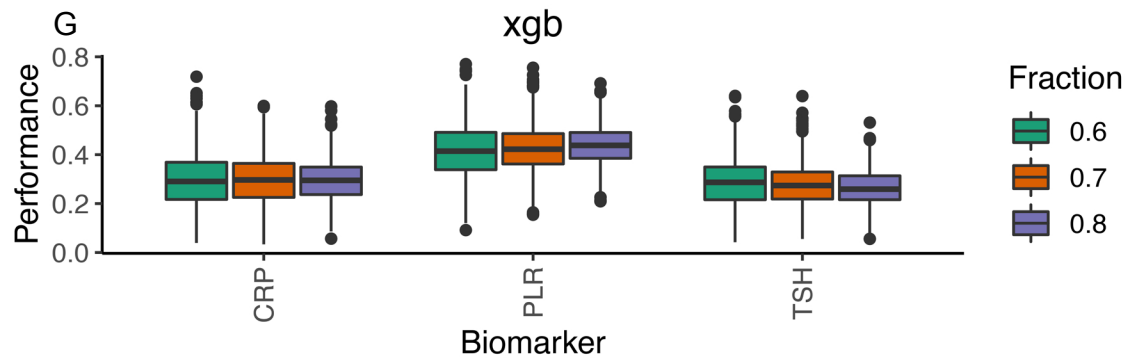

Supplement: Supplementary Figure 3 — Performance of the 3-biomarkers panel evaluated by 7 ML methods. Performance scores were computed by each machine learning method for 1,000 simulations of the training and test datasets at 8:2, 7:3 and 6:4 cohort ratios randomly selected from the combined cohort comprising 1,320 atezolizumab-treated NSCLC patients. The box and whisker plot shows the median (thick black line in the middle of the box), the interquantile range between 75% and 25% (upper and lower end of the box), and 1.5 * upper or lower interquantile range (whiskers), respectively. [file DataSheet_3.pdf]

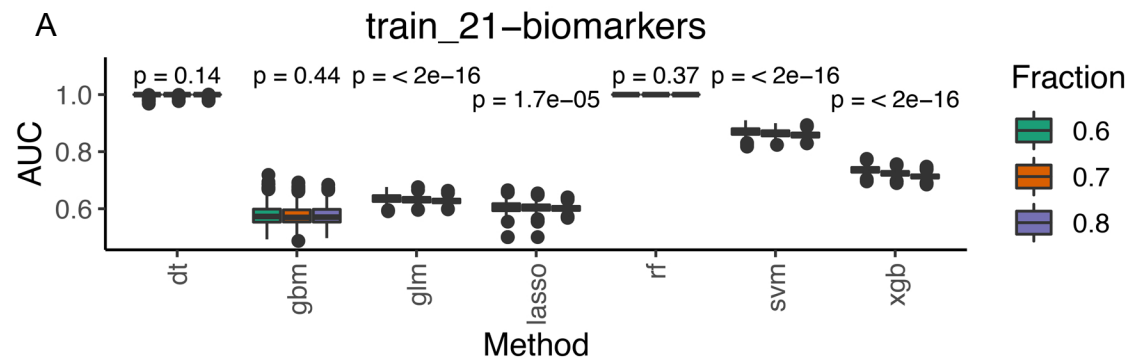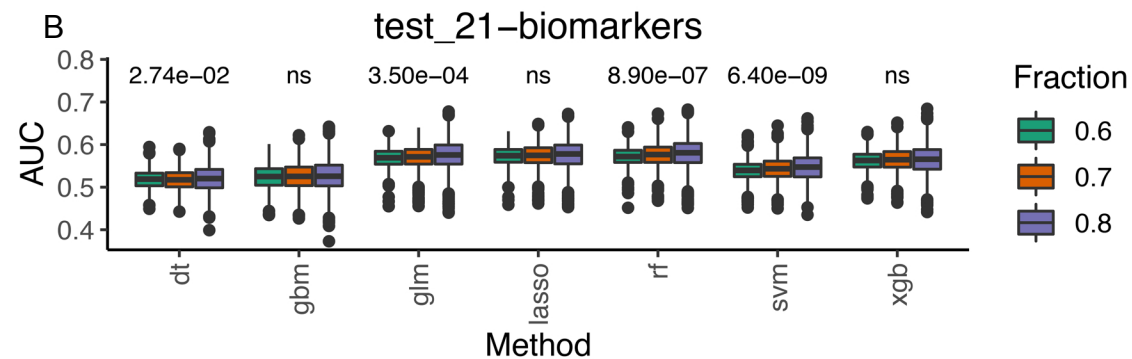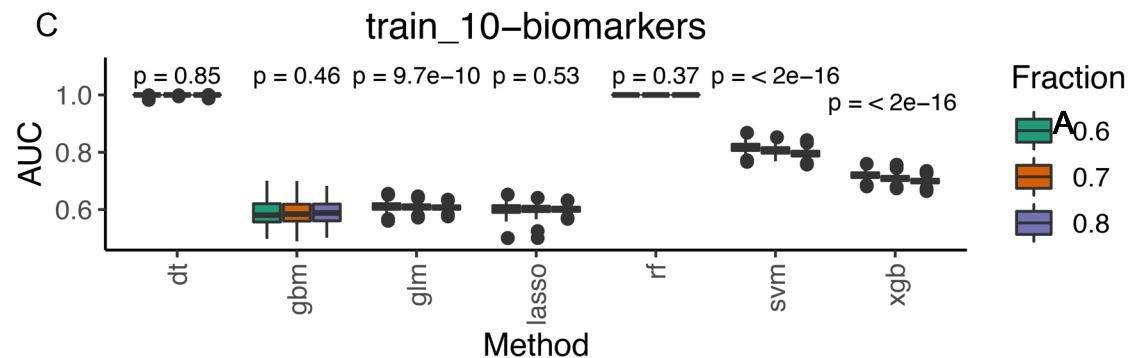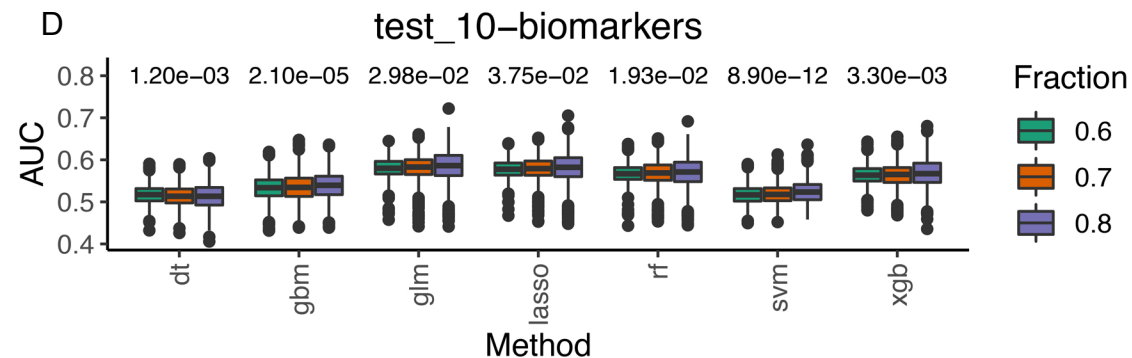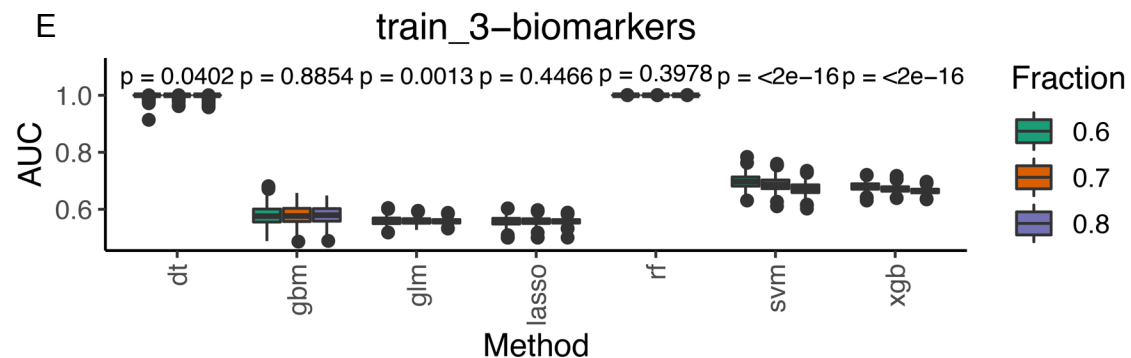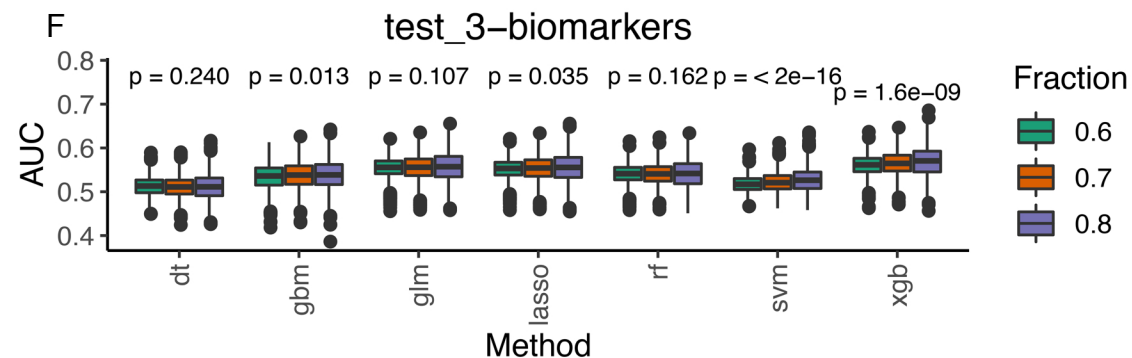

Supplement: Supplementary Figure 4 — AUC distribution of the 3 biomarker panels of this study. AUC was calculated by 7 machine learning methods from 1,000 simulations of the training and test datasets at 8:2, 7:3 and 6:4 cohort ratios randomly selected from the combined cohort comprising 1,320 atezolizumab-treated NSCLC patients. The box and whisker plot shows the median (thick black line in the middle of the box), the interquantile range between 75% and 25% (upper and lower end of the box), and 1.5 * upper or lower interquantile range (whiskers), respectively. [file DataSheet_4.pdf]
