# Supplementary material for: Elucidation of the Application of Blood Test Biomarkers to Predict Immune-Related Adverse Events in Atezolizumab-Treated NSCLC Patients Using Machine Learning Methods
Source: Front Immunol. 2022 Jun 30;13:862752. doi: 10.3389/fimmu.2022.862752 (PMC9284319; doi:10.3389/fimmu.2022.862752)
Supplement: Supplementary Figure 2 — Performance of the 21-biomarkers panel evaluated by the 7 ML methods. Performance scores were computed by each machine learning method for 1,000 simulations of the training and test datasets at 8:2, 7:3 and 6:4 cohort ratios randomly selected from the combined cohort comprising 1,320 atezolizumab-treated NSCLC patients. The box and whisker plot shows the median (thick black line in the middle of the box), the interquantile range between 75% and 25% (upper and lower end of the box), and 1.5 * upper or lower interquantile range (whiskers), respectively. [file DataSheet_2.pdf]

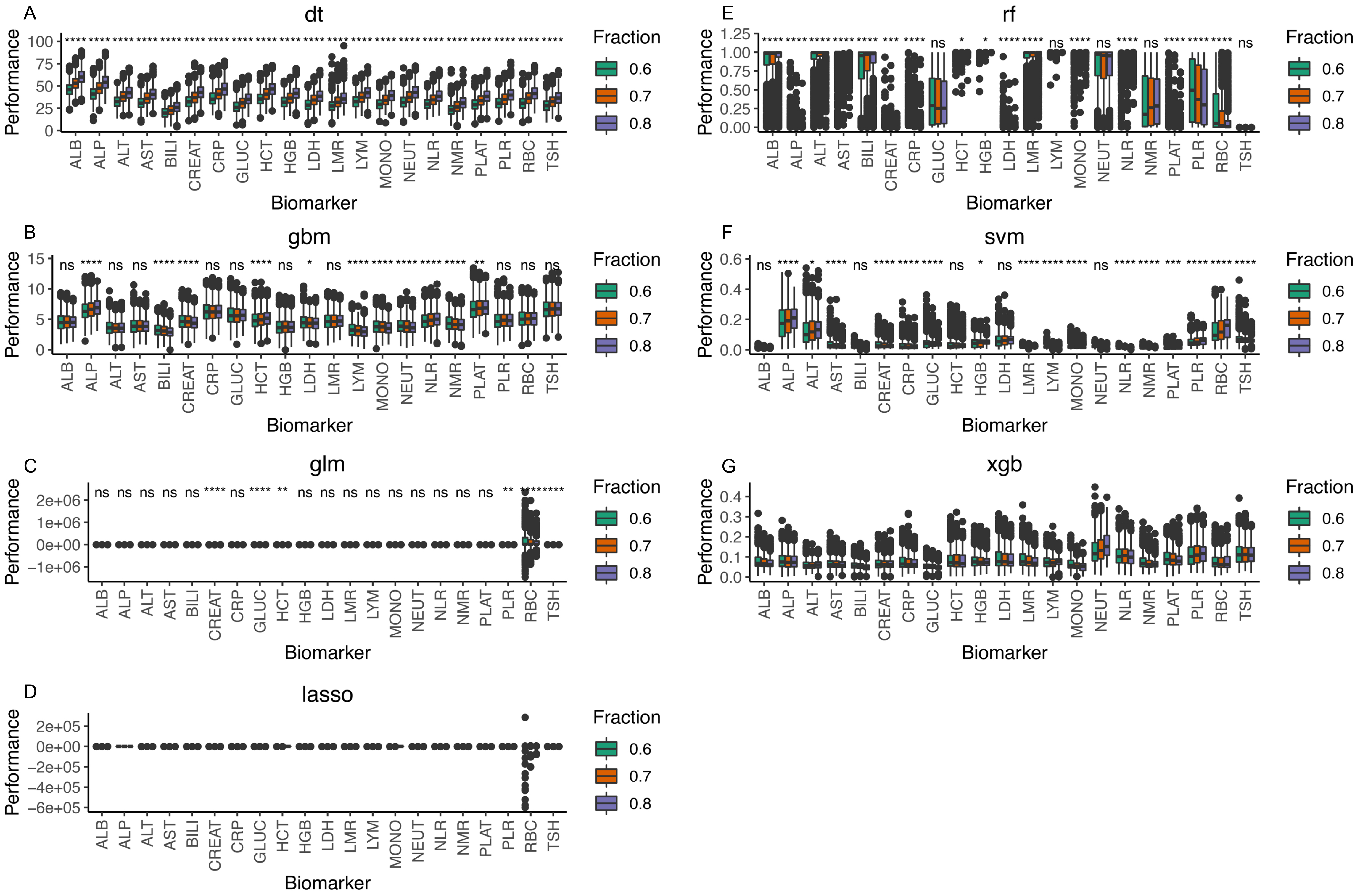

A
dt
B
gbm
C
glm
D
lasso
E
rf
F
svm
G
xgb
Performance
Biomarker
ALB ALP ALT AST BILI CREAT CRP GLUC HCT HGB LDH LMR LYM MONO NEUT NLR NMR PLAT PLR RBC TSH
Fraction
0.6
0.7
0.8
0 25 50 75 100
0 5 10 15
2e+06 1e+06 0e+00 −1e+06
2e+05 0e+00 −2e+05 −4e+05 −6e+05
0.00 0.25 0.50 0.75 1.00 1.25
0.0 0.2 0.4 0.6
0.0 0.1 0.2 0.3 0.4
